# Opening the Curtains on Medical Students' Engagement With Ward Rounds: A Qualitative Study

**DOI:** 10.7759/cureus.93333

**Published:** 2025-09-27

**Authors:** Areen Wazir, Sanjay Pandita

**Affiliations:** 1 Psychiatry, East Kent Hospitals University NHS Foundation Trust, Kent, GBR; 2 Child Health, East Kent Hospitals University NHS Foundation Trust, Kent, GBR

**Keywords:** medical student, student learning, undergraduate medical education, ward round, work-based learning

## Abstract

Introduction

Ward rounds are a key component of medical students’ clinical training, yet there is inadequate research on students’ learning in this setting. Although ward-based learning has been widely studied, research focused specifically on medical students’ experience on ward rounds with explicit utilization of educational theory remains limited. The study addressed this gap through its aims of exploring medical students’ perspectives and engagement on ward rounds, examining how these may change over time, and considering how students' learning experience could be improved, while drawing explicitly upon work-based learning theory. The overarching research question was: How do medical students engage with ward rounds, and what strategies could enhance their educational experience?

Methods

Qualitative exploration was carried out at a large UK medical school. Eleven individual semi-structured interviews, each lasting 30-60 minutes, were conducted over Microsoft Teams with students in the final three years of the program. Transcripts underwent inductive thematic analysis, following Braun and Clarke’s framework, assisted by NVivo software (QSR International, Melbourne, Australia). Social-cultural learning theories shaped topic guide development and were utilized as sensitizing concepts during analysis.

Results

This study suggested that students’ perspectives on, and educational experience of, ward rounds evolve as they progress through years three to five, revealing a shift from learning from doctors to learning to be doctors and highlighting different views on how learning occurs in the workplace. Students appeared to engage more passively than actively on ward rounds, although this decreased as they advanced toward graduation. Despite recognizing the great educational potential of ward rounds, all students expressed some dissatisfaction, which led them to offer suggestions for improvement.

Conclusion

Ward rounds remain an underutilized pedagogy with scope for more meaningful student engagement. Enhancing communication and alignment between medical schools, placement providers, doctors, and students, while adopting a socio-cultural approach, could maximize ward round learning and better bridge the university-workplace gap.

## Introduction

Ward rounds are a routine hospital activity where patients are reviewed, and decision-making regarding diagnosis, management, and discharge is carried out. Since the first recorded ward round in 1660, they have been widely used in medical education for students and junior doctors [1]. Attending ward rounds is an integral part of United Kingdom medical schools’ hospital placements and should offer rich learning opportunities; however, existing literature has questioned their educational value [2]. The quality of teaching and learning in them can be compromised due to several conflicts. First, conducting ward rounds is compared to “walking a tightrope,” balancing the primary purpose of patient care with the additional opportunity for education [3]. Second, differing viewpoints on learning shape how clinician-educators approach this dual task. This can be illustrated by Sfard’s metaphors for learning [4]. "Learning-as-acquisition," rooted in cognitive-behavioral theory, emphasizes the individual learner (recipient) acquiring knowledge and skills from the teacher (provider) [5]. Teaching and learning are seen as distinct activities from working, leading to the conflict articulated above. Sfard’s "learning-as-participation" is grounded in Lave and Wenger’s socio-cultural theory, highlighting meaningful participation in a shared activity. Knowledge is deemed an aspect of practice where the teacher (expert participant) encourages the student (apprentice) to contribute to the activities of a community [6]. This view has the potential to reconcile tensions between service provision and teaching. Third, as per Evans and Guile, medical students may not immediately recognize that the values and logics surrounding knowledge and learning in the workplace are different from those in the classroom and may fail to recognize learning opportunities inherent in work activity [7]. These various conflicts can affect students transitioning from a traditional classroom to a unique workplace environment.

While much research exists on ward-based learning, studies specifically on ward rounds are limited. The available literature on ward round learning seems to largely focus on junior doctors’ experiences, so it may not all be directly transferable to medical students. However, there are key areas to acknowledge regarding potential opportunities and barriers to ward round education. Key areas of ward round learning include clinical knowledge, communication skills, professional skills, and institutional culture [8]. Education during ward rounds is often implicit, embedded in patient care, rather than explicitly taught at the bedside. How students view and engage in ward rounds needs to be further explored. The ward round environment and the senior clinicians on ward rounds are frequently discussed as affecting learning, while the mindset of learners requires further investigation. Traditionally, medical students are onlookers during ward rounds, which is insufficient for maximizing learning. Research suggests that actively engaging learners should be prioritized, but how well this is implemented remains unclear [2,9-11].

Ward rounds are relatively understudied, with few studies aimed at understanding students’ experiences, and existing research lacks thorough utilization of educational theory when viewed through typologies of theoretical visibility in qualitative research by Bradbury-Jones et al. [12]. This study explores medical students’ experiences of ward round learning, explicitly drawing upon work-based learning theory. The aims of this research are to explore student perspectives and engagement in ward round learning, examine how these perspectives and levels of engagement may change over time, and identify ways to improve the educational value of ward rounds. The overarching research question is: How do medical students engage with ward rounds, and what strategies could enhance their educational experience?

This article was presented as a meeting abstract and oral PowerPoint presentation at the AMEE (Association for Medical Education in Europe) conference 2023.

## Materials and methods

Research design 

This research is based on a constructivist ontology and interpretivist epistemology, so the focus is on understanding experiences rather than measurement and rigid explanation. As such, a qualitative methodology was employed to give voice to participant perspectives, allowing exploration of issues beneath the surface of behaviors [13]. The study inclusion criterion was students in years three to five of the MBBS program, as this is the period when they would be expected to take part in ward rounds. Exclusion criteria included students who withdrew consent, those who were not medical students, or those who were not in the final three years of the program. For pragmatic reasons and the potential to provide rich data, recruitment was based on convenience/voluntary response sampling. Digital recruitment posters were circulated through existing medical student year group WhatsApp chats and the online school newsletter, reaching all students in the clinical years of the program. A university email or a link to a Google sign-up form was provided in the advertisement so students could get in touch if they were interested in taking part. Eleven students expressed interest in participating and were subsequently recruited (four intercalating students who had completed the third year at the same medical school where this study was conducted, four students in the fourth year, and three students in the fifth year).

Data collection and storage

Semi-structured interviews (SSI) were selected because of their flexibility; they afford opportunities to ask questions in response to interviewees’ verbal and non-verbal cues, encouraging two-way communication that yields rich data [14]. SSIs were favored over structured interviews, which lack flexibility and offer little opportunity to build rapport between the interviewer and participant. A topic guide was constructed to support the SSI design, highlighting the broad themes supplemented by a list of guiding questions and follow-up questions (Appendices). The major interview questions explored students’ experiences of ward rounds, the activities undertaken during them, changes in ward round experiences over time, factors influencing learning, and levels of satisfaction with ward round education, including potential areas for improvement. The topic guide helped maintain consistency of themes covered between interviews; however, its flexibility enabled adaptation in response to participants’ answers. It was created primarily by drawing on available literature on ward rounds, as well as brief informal conversations with a few students prior to starting this research, who had expressed some dissatisfaction with ward rounds as a pedagogy. Feedback on the topic guide from a professor of medical education and validation from the ethics committee helped ensure relevance and rigor.

One-to-one SSIs were conducted between February and April 2023, using a topic guide that drew upon work-based learning theory. The interviews lasted between 30 and 60 minutes on Microsoft Teams. The online format was selected due to the convenience of conducting the interview from a location of choice and the combined recording and auto-transcription feature offered. These interviews were recorded, and an intelligent verbatim transcription was carried out, making the transcripts more readable and allowing familiarity with the data before analysis. Anonymized transcripts were stored securely on AW’s Microsoft OneDrive, and recordings were stored securely on MS Teams via SharePoint with a technical safeguard in place on a password-protected account. These recordings were deleted at the end of the research period.

Data analysis 

Inductive thematic analysis of transcripts was carried out with the assistance of NVivo software (QSR International, Melbourne, Australia), following Braun and Clarke’s six-step framework [15]. The six phases of the analysis are as follows chronologically: data familiarization, generating initial codes, searching for themes, reviewing themes, defining and naming themes, and finally, producing the report. Data familiarization involves transcribing the data, reading through the transcripts, and noting initial codes. Generating initial codes builds on this by identifying meaningful segments in the data, developing initial codes, and collating data relevant to each code. Searching for themes involves looking for patterns and similarities between codes and linking them together to form preliminary categories, followed by bringing together categories to form preliminary themes. Next is reviewing themes, where the themes, categories, and codes are checked to ensure they work together and accurately represent the data to produce a thematic map. Once reviewed, defining and naming themes involves refining themes and allocating names to finalized themes. Finally, producing the report entails finding quotes from interviews to highlight each theme and relating the analysis back to the research question and literature to produce a report [15]. The steps for analysis were led by AW, but coding and finalized themes were also reviewed by a professor of medical education to ensure coding rigor and consistency in the analysis stage. Debriefing from two senior professionals with experience in medical education, who were uninvolved with this research, also provided feedback on coding and theme refinement, which enhanced the quality of finalized themes. Thematic analysis was deemed appropriate as it offers a powerful approach for understanding people’s experiences and provides a more accessible, structured method to summarize characteristics of large data sets. It is useful for highlighting similarities and differences between participant perspectives and generating unanticipated insights [16]. Following the analysis, relationships between codes, categories, and themes were developed, with the finalized four overarching themes shown in Table 1. These relationships guided the discussion.

**Table 1:** Relationship between codes, categories, and themes

CODE	CATEGORY	THEME
Greater trust in students	Change in doctors’ attitudes	Evolution in medical students’ educational experience on ward rounds from year three-five
Offering students more active roles on ward rounds		
Higher expectations of students		
Greater motivation to understand the job	Change in students’ attitudes	
Greater drive to participate actively in ward rounds		
Greater confidence and expectations of oneself		
More hands-on and active opportunities	Change in activities	
Opportunities are similar, but students do more of them		
Having a more valuable role in helping the ward round team		
Doctors providing teaching by telling students information	Learning from doctors	Student perspectives on purposes of ward rounds
Students observing and listening		
Preparation for exams		
To prepare for Foundation year 1	Learning to be doctors	
Opportunity to clerk patients		
Getting familiar with the staff and their responsibilities		
Learning admin skills		
Contextualize knowledge		
For patient care rather than student learning	Other	
Curriculum requirement		
Observing activities	Passive	Students’ engagement on ward rounds
Listening to doctors and discussions		
Doing mundane tasks, e.g., closing curtains		
Engaging in case discussions	Cognitively active	
Interpreting scans/blood results/ECGs		
Examinations	Physically active	
History taking		
Presenting patient case		
Writing up patient notes		
Procedures		
More guidance and information for doctors on what students will be there on ward rounds and how to engage them	Targeting doctors on ward rounds	Students’ ideas on strategies to improve ward round education
Doctors should offer more active learning opportunities		
Designated ward round doctor for each student		
More student guidance on how to make the most out of ward rounds	Targeting students	
Reduce student-ward ratio	Targeting the ward round structure	
More structure to ward round learning (having a goal to achieve for each ward round)		
Greater emphasis on active learning through participation		

Ethical considerations

Ethical approval was obtained from the Queen Mary University of London, Institute of Health Sciences Education, Internal Peer Review and Ethics Committee with reference IPREC221207.WAZ. Ethical principles were followed in compliance with the Declaration of Helsinki. Participation was voluntary, and informed consent was obtained before recruiting students. The participant information sheet explained full details of the study, including what data were collected, how the data were stored, used, and destroyed, as well as information on how to withdraw from the study and whom to contact for questions. This helped potential participants decide whether they wanted to take part. If they agreed, they read, signed, and emailed the consent form, and consent was reaffirmed verbally before starting the interviews.

Maintaining privacy and participant confidentiality was paramount. Therefore, interviews were conducted with only the participant and AW on MS Teams, which uses mutual transport layer security to authenticate both parties and encrypt all call traffic. The interview recordings were stored securely and deleted promptly after transcription. Additionally, participant details were anonymized; for example, students were assigned labels such as "Student A," and any identifiable information, such as the names of hospital placements, was removed from the transcripts.

## Results

The analysis generated four key themes.

Theme 1: “drag-along” (Student B) to the hospital assistant

The analysis reveals an evolution in medical students’ experiences of ward rounds as they progress toward qualifying, reflected in reported shifts in both their own and doctors’ attitudes. As medical students move from year three to five, doctors appear to have greater trust in them and involve students more in ward round practices. There is also a shift in student mindset, as fifth years appear more confident and motivated to seek opportunities to contribute to ward rounds, linked to their greater placement experience and realization that they will be doctors imminently.

*"(Fifth year) you’re feeling more confident…but you also know you’re gonna be a doctor soon, so you’re getting yourself more involved” *(Student B).

*“Bigger level of trust and they (Year Five) have to start working next year…doctors want to give them more responsibility” *(Student A).

This combined shift in attitudes manifests as a change in student engagement and their ward round experience, from being largely overlooked and passive in third year to becoming more valuable, active members of the ward round team in fifth year.

*“Third year was a bit more passive…just observe…fourth year was a bit more hands on…they’ll ask for your input” *(Student D).

*“[Year Five is] more stepping into the shoes of a doctor…doing bits of examinations, presenting cases, getting you more involved in discussions as opposed to just listening” *(Student K).

Theme 2: learning from doctors or learning to be doctors

The second theme highlights shifts in the way students view the purposes of attending ward rounds. Some view the purpose as learning from doctors by taking a backseat role, listening and observing while the doctors teach them:

*“It’s…for me to learn. Whereas I’m not actually benefiting the team” *(Student C).

Meanwhile, others see the purpose as learning to be doctors through engaging in activities that allow them to step into the shoes of a doctor and contribute to ward rounds: 

*“Help see patients or help with some of the jobs that crop up in the ward round” *(Student J).

There is some suggestion that the purpose of ward rounds for students evolves as they progress through their clinical years, shifting from learning from doctors to learning to be doctors.

*“Purpose as you edge more towards the end is not just…trying to learn to pass exams but…more about learning how to do the job…(in fifth year) placements are called hospital assistantships” *(Student J).

*“Onto fifth year, I’d be focused more on what are the junior doctors doing…thinking of it more like an apprenticeship” *(Student C).

Theme 3: passive and active engagement

The analysis illuminates a range of ward round activities that students engaged with, categorized as passive (observing, listening, and performing mundane tasks, e.g., opening/closing curtains), cognitively active (case discussions, interpreting scans, blood results, or ECGs), and physically active (examinations, history taking, presenting cases, procedures, and writing patient notes). There was a shared agreement that ward rounds largely involved passive activities, although this decreased as students advanced toward qualifying. Active learning opportunities were greatly valued as they allowed contextualization of knowledge and skills and enabled students to step into the shoes of a doctor.

*“Best moment” *on ward rounds:* “One person history, one person examination in front of the team and that got rid of my…stage fright...he (consultant) appreciated us and gave feedback” *(Student H).

*“When you talk about a case…it helps consolidate knowledge and when you can just contextualize your textbook knowledge on a patient, it's so helpful” *(Student A). 

Meanwhile, passive ones also offered potential learning, e.g., communication, empathy, and teamwork

*“Just observing and spectating…you’re still getting some form of learning” *(Student D).

*“...How to communicate with patients…through good and bad examples” *(Student B).

Theme 4: improving ward round education

**Figure 1:**
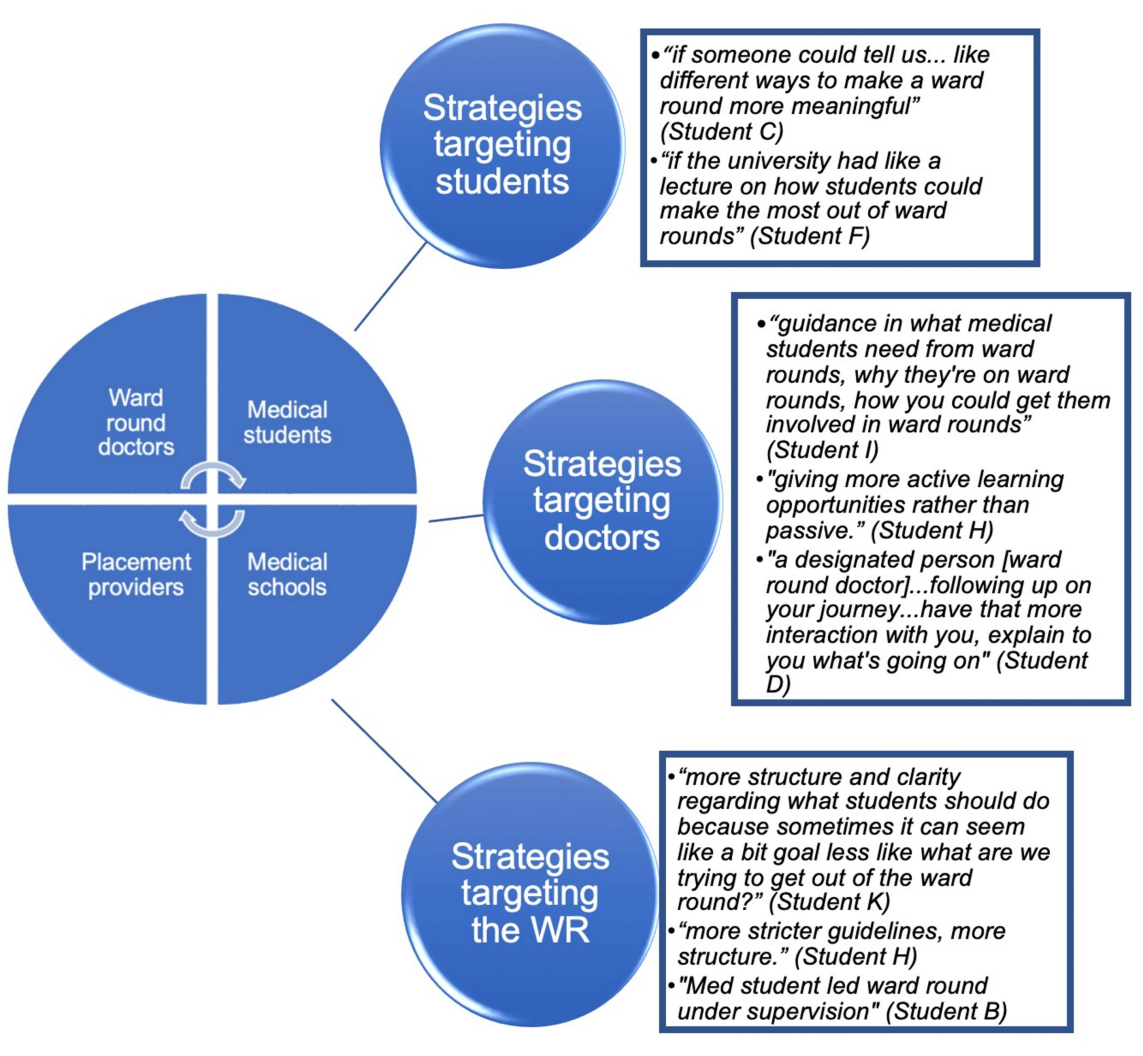
Strategies for improving students’ learning on WR WR, ward rounds Image Credits: Areen Wazir

There remains a degree of dissatisfaction with ward round education across all participants, with several classifying it as “hit or miss.” Students cited the wide variability in the educational value between ward rounds due to an interplay of several factors: doctors on ward rounds, students’ approach, ward round environment, etc. Upon amalgamating participants’ ideas on improving ward round education, the root of all suggestions required greater communication between the four stakeholders. Participants’ ideas were divided into three groups (Figure 1).

## Discussion

In summary, the analysis suggests that students’ experience of ward rounds evolves as they progress from years three to five, reflected in the change in attitudes of both doctors and students. There are also shifts in students’ perceptions of the purposes of ward rounds over this period. Ward round activities can be categorized as passive or active, with active ones further divided into cognitive and physical activities. Overall, students view ward rounds as a fickle pedagogy influenced by various factors and have offered a range of strategies to improve their learning experience.

There appears to be a shift from cognitive-behaviorism to more socio-cultural approaches in how students view and experience ward rounds over time. This can be understood through Sfard’s metaphors, each offering something the other cannot provide [4]. Students who view ward rounds as a place to “learn from doctors” may be holding onto classroom logics of learning focused on acquisition [7,17]. Acquisition is the dominant view of learning, reflected in behavioral (focused on skill acquisition and role modeling) and cognitive (focused on knowledge acquisition through transmission or constructivism) schools of thought [5]. They sometimes overlook the context in which learning occurs and forget that competent individuals can form incompetent teams [18]. A tunnel-visioned view of learning solely as acquisition can result in students missing valuable educational opportunities.

**Figure 2:**
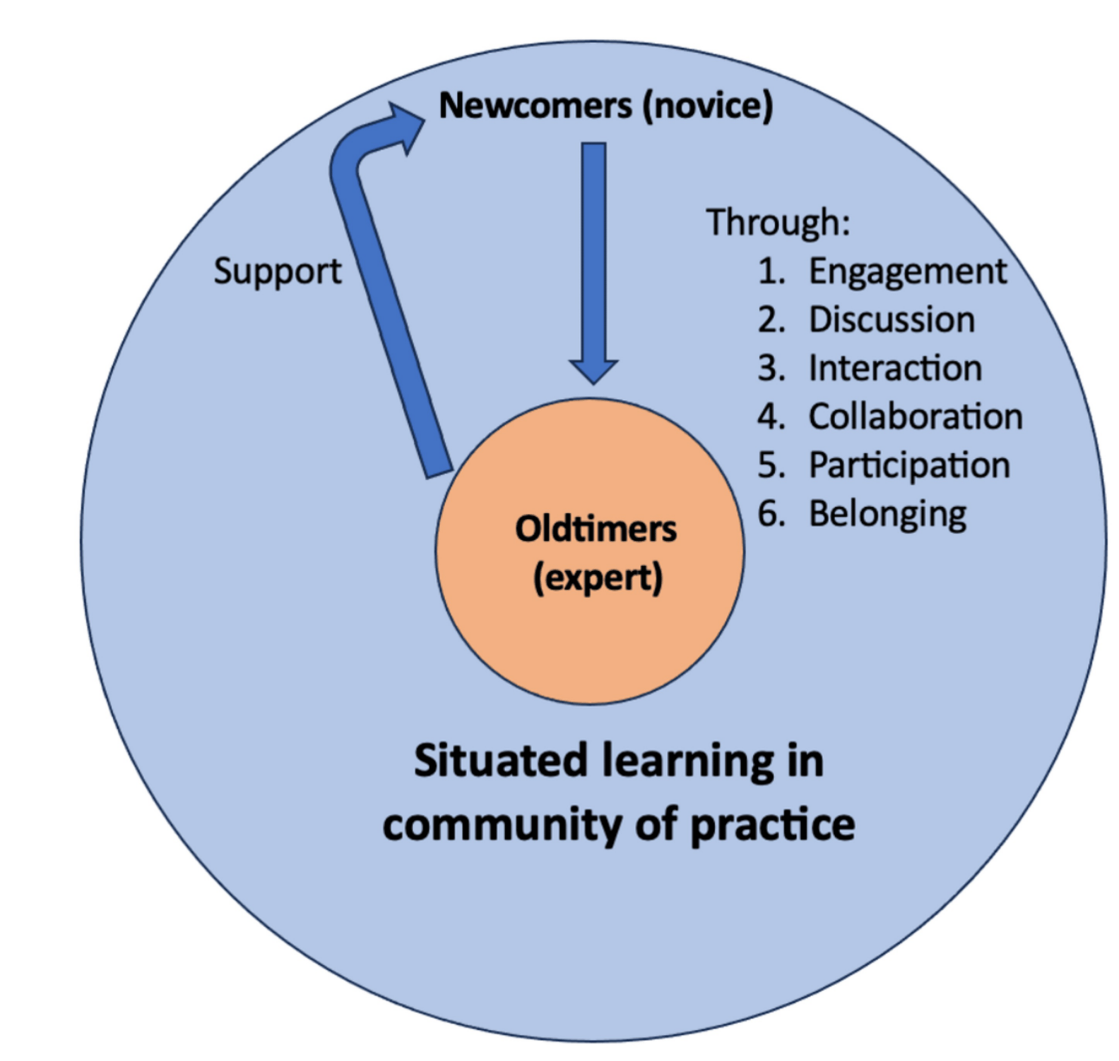
Legitimate peripheral participation Figure created by Areen Wazir, adapted from Hindi et al. (2022) under the Creative Commons Attribution 4.0 International license [23]

Those who see ward rounds as opportunities to “learn-to-be-doctors” may recognize that the logics of the workplace are different [7]. Their views and accounts of learning mirror legitimate peripheral participation (LPP) in communities of practice (CoP) in Lave and Wenger’s socio-cultural theory (Figure 2). Socio-cultural theory postulates that learning and working are a partnership and that people learn through “situated learning in CoP” [6]. LPP conveys that newcomers are invited into CoP by old-timers and are engaged in increasingly meaningful activities and conversations that enable them to become more active participants [6]. Adopting a socio-cultural perspective supports the view that the purpose of medical education in clinical years should largely encompass the process of becoming a doctor and belonging to a healthcare team [19]. Several commentators, such as Swanwick and Lingard, support this position, although more often in postgraduate years [20,21].

The concepts of LPP and CoP do not fully explain engagement on ward rounds. Billet argues that workplace affordances and personal agency contribute to the quality of learning experiences in the workplace, so learning on ward rounds is an interaction between an agentic individual’s mind and a socially constructed CoP [22]. This could explain how students’ mindsets and approaches toward ward rounds influence their learning. Equal consideration should be placed on staff offering opportunities to actively participate and on how students choose to engage with them.

Although active ward round learning opportunities focused on “participation” were highly valued, passive ones focused on “acquisition” should not be discarded. Observational learning is important, given that patient safety prevails over training. A study [24] by Stegmann et al. showed that vicarious learning led to greater knowledge of doctor-patient communication than enactive learning. Meanwhile, a study [25] by Prado et al. concluded that active methodology produces better results than traditional, more passive methods in ward-based contexts. Embracing metaphorical pluralism with greater emphasis on participation seems prudent for ward round learning. Participants viewed active learning positively as it allows contextualization of knowledge and skills and enables students to experience some responsibilities of a doctor. For example, students cherished the rare opportunity to clerk patients on ward rounds, which links to learning through meaningful participation in CoP.

Evans and Guile argue that workplace learning involves the important process of re-contextualization, in which knowledge and skills are adapted and applied to unique contexts at work [7]. Contextualization enables learners to engage in deep, meaningful learning, make connections between theory and practice, and link information to broader social, cultural, and political contexts. In workplaces, knowledge is ingrained in routines, protocols, and artifacts, and the primary obstacles revolve around learning to participate in workplace activities [17]. Students require support bridging university and workplace environments to foster the chains of re-contextualization. To extract the full educational potential from ward rounds, students and educators must understand the limitations of mismatching these logics between the two contexts.

Strategies targeting students 

Students believed it would be beneficial if they were provided guidance on how to optimize ward rounds for their learning. This could be done by encouraging students' understanding of workplace learning logics. A study [26] by Quilligan suggested that when students changed their understanding of knowledge and that learning was about becoming a doctor, clinicians offered more participatory opportunities. Additionally, equipping students with tools to enhance personal agency, e.g., the STIC framework, is helpful, giving students some control over their learning on ward rounds, transforming chaotic events into more focused learning scenarios [27]. 

Strategies targeting doctors

Providing more guidance to ward round doctors on how to engage students was important to participants. Doctors should give students a role and actively involve them in ward round practices to help them apply classroom-learned knowledge in the workplace, enabling meaningful contributions to ward rounds and supporting patient care. It is important to integrate context in teaching and learning to facilitate the classroom-workplace transition [28]. As such, doctors should ensure that information relayed to students on ward rounds is contextualized.

Strategies targeting the ward round structure

Many students agreed that increased structure in ward round learning would be beneficial. Having clear educational goals to achieve by the end of the ward round would encourage students and doctors to work together to achieve them. This idea is supported by a study [10] by Noorani on postgraduate trainees, which concluded that impromptu learning without clear goals becomes uninteresting, overwhelming, and offers little benefit for ward round learning. Additionally, students wanted more active learning on ward rounds, with a few suggesting taking the lead when deemed appropriate by doctors. This aligns with the "upside-down" ward round, a technique in postgraduate settings where junior and senior doctors switch roles, allowing the junior to lead [29]. This approach could be applied in undergraduate education by giving students opportunities to conduct patient encounters on ward rounds under supervision and receive feedback.

Recommendations

Overall, the following recommendations could enhance learning on ward rounds: providing students with clear guidance on how to utilize ward rounds for learning by embracing workplace learning logics and offering frameworks to enhance personal agency; providing doctors with clear guidance on how best to engage students by promoting active learning opportunities such as case discussions, clerking patients, and interpreting investigation results; encouraging doctors to help students embrace workplace logics, make learning opportunities explicit, and contextualize experiences on ward rounds; implementing the "upside-down" ward round technique where deemed safe by doctors; and creating structure in ward round learning through well-defined, measurable goals for students to achieve by the end of each ward round, potentially incorporating these goals into logbooks, such as conducting a history or examination and presenting on the ward round.

Strengths and limitations

The strengths of this study lie primarily in the collection of rich data. Eleven SSIs lasting 30-60 minutes with students from different clinical years enabled data saturation, demonstrating robust data collection, content validity, and qualitative rigor. Additionally, rigorous thematic analysis was employed to identify patterns and themes, and educational theory was thoroughly utilized. It is also necessary to acknowledge the limitations. Non-probability sampling threatened external validity, as it may not be representative of the entire target population, and all participants studying at the same institution limit the transferability of results. As the data collection and analysis were led by AW, there was a risk of researcher bias. This was mitigated by constructing a topic guide driven primarily by available literature rather than personal perceptions, seeking feedback on the topic guide, anonymized transcripts and analysis from a professor of medical education, engaging in peer debriefing, and receiving training in qualitative interview techniques to minimize interviewer bias.

## Conclusions

Ward rounds are an underutilized pedagogy, holding significant potential for learning and a clear scope for more meaningful student engagement. They are arguably the most crucial element of the day for doctors and patients, acting as a blueprint for the rest of the day. Ironically, they are not always beneficial for medical students. Improving undergraduate ward round education requires better communication and alignment between medical schools, placement providers, doctors, and students, along with the adoption of a largely socio-cultural approach. Emphasizing "learning-to-be-doctors" over "learning-from-doctors" will enable students to engage more meaningfully.

## Appendices

Topic guide

This topic guide is flexible, as questions may be molded and additional questions may be asked depending on the answers given by the interviewees.

INTRODUCTION:

→ Thank the participant for their time

→ Take consent

→ Reaffirm what the research will entail

→ Check if the participant has any questions

INTERVIEW:

→ Personal background

- Year of study

- Whether on a GEP/undergrad course

→ Experience of ward rounds

- First of all, can you tell me about where you have experienced ward rounds so far in your course?

- What do you see as the purpose or purposes of attending ward rounds as a medical student? Do you see that changing as the course progresses?

- How satisfied are you with ward rounds as an educational experience at medical school?

- How useful/not useful do you find ward rounds?

 * Why?

- Please share an experience you’ve had on ward rounds that positively impacted your learning? What does this form of teaching offer that you don’t get elsewhere?

- What do you think is your role during ward rounds? What influences that, do you think?

→ Activities on ward round

- Can you tell me more about the things you do/have done during ward rounds? Which do you find most/least useful? (things that may be brought up by the interviewee):

  * Clerking patients

  * Presenting patient cases

  * Interpreting scans/blood results

  * Ward rounding up patient notes

  * Case-based discussions

  * Other?

- What skills do you think you learn whilst on a ward round? (things that may be brought up by the interviewee):

  * Hard skills like examination/history taking

  * Soft skills like communication/empathy/teamwork/leadership

  * Other?

→ Changes in ward round experience

- For 4th or 5th years: How would you compare your experience on ward rounds this year compared to when you were in year 3/year 4?

- For 3rd or 4th years: Do you think medical students’ experience and role on ward rounds change as they progress through medical school?

  * In what way?

  * Why?

→ Factors impacting learning on ward rounds

- What factors do you think limit your learning on ward rounds? (e.g., ward environment/lack of support or interest from doctors on the ward round/lack of knowledge or personal motivation/feeling like you don’t belong, etc.)

→ Changes to ward round education for medical students

- Overall, do you think ward rounds on placement accommodate well for medical student learning?

  * Why?

- What opportunities or changes would you like on ward rounds to maximize your learning?

WRAP-UP:

→ Is there anything else I should have asked but didn’t?

→ Do you have any other final comments on anything we discussed?

→ Thank the participant
